# Editorial: Brain adaptations to exercise in health and neurodegenerative diseases: considerations and future perspectives on the underlying mechanisms

**DOI:** 10.3389/fphys.2025.1770850

**Published:** 2026-01-07

**Authors:** Ida Cariati, Roberto Bonanni, Pierangelo Cifelli, Giovanna D’Arcangelo

**Affiliations:** 1 Department of Systems Medicine, “Tor Vergata” University of Rome, Rome, Italy; 2 Department of Human Science and Promotion of Quality of Life, San Raffaele Open University, Rome, Italy; 3 Centre of Space Bio-Medicine, “Tor Vergata” University of Rome, Rome, Italy

**Keywords:** brain adaptations, exercise, exerkines, mental health, neurodegeneration, neuroprotection, physiology, training

Physical exercise is known to have numerous benefits for the central nervous system (CNS), improving cognitive function and counteracting the onset and progression of neurodegenerative diseases. However, the molecular mechanisms underlying these positive effects have not been fully elucidated, highlighting the need for further research in the field of exercise physiology (Bonanni et al., 2024).

The available evidence suggests a multi-pathway effect of exercise, capable of influencing neuronal redox status, neurotrophic factor synthesis, neurogenesis, synaptic transmission and plasticity, as well as cerebral blood flow and oxygenation (Izawa et al., 2024; Rong et al., 2025; Cariati et al., 2025). Particularly, Xu et al. investigated the role of physical exercise in modulating the mitochondrial mechanisms involved in the pathogenesis of Parkinson’s disease, highlighting how alterations in mitochondrial biogenesis, mitophagy and neuronal redox balance contribute to the degeneration of dopaminergic neurons. Specifically, exercise appears to restore mitochondrial homeostasis by regulating the production of reactive oxygen species (ROS), activating neuroprotective exerkine-mediated signals and increasing the expression of neurotrophic factors, supporting its potential as a complementary non-pharmacological intervention in the management of the disease. These observations are consistent with previous studies that have shown that exercise stimulates the production of neurotrophins, key molecules in protecting neurons and counteracting neurodegenerative processes (Lu et al., 2024; Gholami et al., 2025). Among the exercise-induced neurotrophins, brain-derived neurotrophic factor (BDNF) has attracted considerable interest both for its role in neuronal survival and for its involvement in depressive disorders. In this regard, the systematic review with meta-analysis by Fang et al. demonstrated that exercise interventions can significantly alleviate depressive symptoms in haemodialysis patients, highlighting how protocols characterized by high adherence to the American College of Sports Medicine (ACSM) guidelines are more effective than those with low or uncertain adherence.

The neuroprotective effect of exercise was confirmed by Cariati et al., who found ultrastructural and functional improvements in the hippocampus of mice undergoing aerobic training, suggesting a positive effect on cognitive function (Cariati et al., 2021). However, it remains unclear which type of exercise is optimal for maximising benefits to the CNS. In this context, Lingling et al. demonstrated that low-intensity aerobic exercise can improve executive performance and modulate cortical network dynamics in healthy young adults. Specifically, the authors highlighted an optimization of functional connectivity at the parietal level and an enhancement of interactions between motor and sensory areas, suggesting a key role for low-intensity exercise in motor learning processes and cortical plasticity mediated by the primary motor cortex. On the other hand, Allison et al. have provided interesting evidence on the role of resistance training in improving cerebrovascular function, representing an ideal tool for promoting brain health during aging and counteracting the development of Alzheimer’s disease and related dementias. In agreement, Elbanna et al. found cerebrovascular adaptations in response to physical and mental tests in elderly people with mild amnestic cognitive impairment and cognitively normal individuals, suggesting the possibility of combining physical and mental activity to improve cerebral perfusion and oxygenation and delay the onset of cognitive impairment.

Finally, La Greca et al. showed how specific verbal instructions during the execution of the vertical drop jump can optimize performance and reduce impact during landing in young volleyball players, highlighting how simple interventions can modulate neuromuscular activity and cerebro-motor connections, with possible indirect implications for CNS health and injury prevention.

Overall, the evidence gathered in this Research Topic represents valuable pieces that enrich the complex mosaic of research on physiological adaptations to physical exercise. It is essential to renew the interest of exercise physiologists to encourage the development of clinical and pre-clinical studies aimed at exploring the mechanisms underlying the beneficial effects of exercise on the health of the CNS and the entire organism.
